# Concerns about cell therapy for intervertebral disc degeneration

**DOI:** 10.1038/s41536-022-00245-4

**Published:** 2022-09-06

**Authors:** Baogan Peng, Yongchao Li

**Affiliations:** Department of Orthopaedics, The Third Medical Center, General Hospital of the Chinese People’s Liberation Army, Beijing, China

**Keywords:** Diseases, Neurological disorders

Low back pain is a very common symptom in people all over the world, which is now the leading cause of disability worldwide^1,2^. Although the specific cause of low back pain is rarely determined, lumbar intervertebral disc degeneration is considered to be the main cause of low back pain^3^. So far, neither conservative treatment nor surgical treatment can prevent or at least slow down the degenerative process^4,5^. For this reason, regenerative medicine, the repair of degenerate discs by intradiscal injection of exogenous cells, is emerging as a promising approach^4^. However, due to its distinctive structure and function, disc presents unique characteristics: largely avascular, hypoxia, low pH, high osmotic pressure and high mechanical load^4,6,7^. This situation establishes an adverse microenvironment for resident cells and delivered exogenous cells, which limits the effect of cell therapy^4,5^. In addition, there are still other considerable challenges in the entire translational spectrum of cell therapy, including the lack of guidelines for disease classification and patient stratification, as well as a marked lack of understanding of the characteristics of neural distribution, cell fate, and long-term prospects for disc regeneration in the context of cell therapy. In this comment, we will discuss the key issues mentioned above in disc cell therapy.

## Intervertebral disc degeneration and cell therapy

**Fig. 1:**
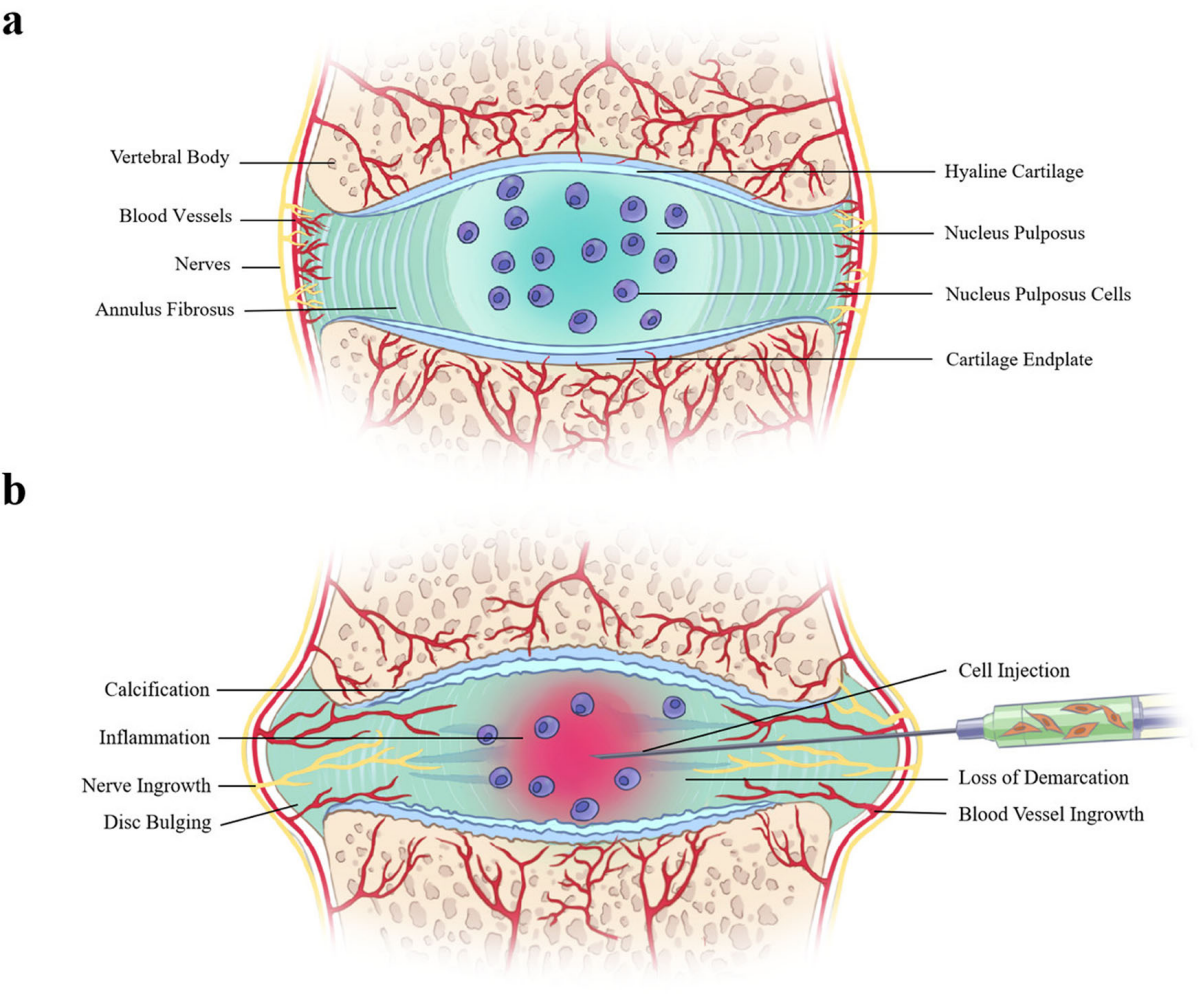
Schematic showing a healthy intervertebral disc and a degenerative intervertebral disc with intradiscal cell injection. **a** Healthy intervertebral disc is composed of annulus fibrosus, nucleus pulposus and cartilage endplates; **b** Degenerative disc shows loss of border between annulus fibrosus and nucleus pulposus, reduction of nucleus pulposus cells, calcification of cartilage endplate, and ingrowth of blood vessels and nerve fibers into the inner layer of degenerative disc.

The intervertebral disc is a complex cartilage structure whose function is to resist biomechanical loading during spinal movement. It is composed of a highly viscous nucleus pulposus surrounded by a thick fibrocartilage outer ring, annulus fibrosus, and sandwiched by cartilage endplates below and above (Fig. 1a). Intervertebral disc cells actively regulate their metabolic activities in a paracrine and/or autocrine manner through a variety of substances, including cytokines, enzymes, enzyme inhibitors, and growth factors. The degeneration of the intervertebral disc is characterized by a decrease in the number of viable cells, especially the number of nucleus pulposus cells, and the decline of their function, resulting in the loss of extracellular matrix (ECM), especially proteoglycans^8^. As the disc degenerates and becomes more dehydrated, the lamellar structure of the annulus fibrosus becomes disorganized and the disc loses its structural integrity^9^. Cartilage endplates tend to calcify, reducing nutrient delivery to cells^9^. With inflammation, blood vessels, and nerves may grow into the inner layer of the annulus fibrosus and nucleus pulposus (Fig. 1b), which correlate to the low back pain^7,10^. These pathophysiological changes will lead to the loss of mechanical tension of the annulus fibrosus and the pressure of the intervertebral disc. Therefore, the ability to maintain or reconstitute ECM by increasing the number of viable cells in the degenerate disc and altering the balance between synthesis and degradation is an emerging therapeutic strategy^5^. At the same time, cell therapy can alleviate the pain of disc origin through immune regulation and inhibition of inflammation^8,11^.

Cell therapy for disc degeneration involves the delivery of viable cells to the nucleus pulposus^12–14^ (Fig. 1b), annulus fibrosus^15,16^ or systemic application^17,18^, either alone or in combination with biomaterial scaffolds and carriers^5^, making it possible to repopulate and repair degenerate discs, or at least modulate the degenerate microenvironment^8^. Various cell types, such as intervertebral disc-derived cells^19–26^, chondrocyte-like cells^27,28^, mesenchymal stromal cells (MSCs)^13,29–35^, olfactory^36^, embryonic^37^, hematopoietic^38^, or induced pluripotent^39–41^ stem cells have been used for regenerative therapy of degenerate discs in basic and/or clinical studies (Table 1). Among these cells, MSCs represent a particularly attractive option and have been widely used in regenerative medicine owing to their easy preparation, self-renewal ability, multilineage differentiation potential, and anti-inflammatory and immunosuppressive properties^8^. This proof of concept has been confirmed by a large number of preclinical studies and several early clinical trials, which provide encouraging results in regenerative effect and reducing low back pain, respectively^42^.

**Table 1:** Cell types proposed for intervertebral disc regeneration.

Cell type	Cell source	Refs.
Intervertebral disc-derived cells	Notochordal cells	^19,24^
	Nucleus pulposus cells	^25,26^
	Annulus fibrosus cells	^20^
	Disc-derived chondrocytes	^21–23^
Chondrocyte-like cells	Articular cartilage-derived chondrocytes	^28^
	Auricular cartilage-derived chondrocytes	^27^
Mesenchymal stromal cells	Adipose-derived mesenchymal stromal cells	^31,32^
	Bone marrow-derived mesenchymal stromal cells	^13,35^
	Umbilical cord-derived mesenchymal stromal cells	^33,34^
	Synovial-derived mesenchymal stromal cells	^29^
	Nucleus pulposus-derived mesenchymal stromal cells	^30^
Stem cells	Olfactory stem cells	^36^
	Embryonic stem cells	^37^
	Hematopoietic stem cells	^38^
	Induced pluripotent stem cells	^39–41^

## Which patients are suitable for cell therapy?

One of the most difficult questions yet to be answered is related to patient selection^8,9^. Which patients are most suitable for cell therapy? When considering the clinical success of cell therapy, it is very important to determine the status of intervertebral disc and evaluate the applicability of cell therapy. Patients come to the clinician because of back pain, not because they are worried about disc degeneration^9^. In fact, many patients with severe disc degeneration have no symptoms. Therefore, back pain rather than disc degeneration should be a clinical goal. In most cases, it is not known whether low back pain is caused by intervertebral disc. Other structures, such as facet joints and sacroiliac joints, may also be involved, so even if the degenerate disc is completely regenerated, the pain may not be cured^9^.

Magnetic resonance imaging (MRI) can well reveal the degeneration and degree of disc degeneration, but it cannot distinguish which degenerative disc is a painful disc^5^. With the increase of age, the loss of proteoglycans in intervertebral disc gradually increases. Therefore, ~10% of intervertebral disc at the age of 50 and ~60% of intervertebral disc at the age of 70 degenerate seriously^43^. This leaves a basic question, that is, what is symptomatic pathology and what is only age-related disc degeneration. Provocative discography now is a diagnostic option. With the emergence of intervertebral disc regenerative medicine, lumbar discography is more and more widely used in pre-injection planning^12,32,44^. However, lumbar discography has been discredited and may indeed cause further disc degeneration^45^. Thus, there is no reliable method to determine which disc is the source of pain. It is urgent to develop new tools to diagnose painful discs requiring cell therapy^5^. New, advanced imaging tools for this purpose are currently under development, including MRI techniques such as T1ρ and T2 relaxation times, as well as chemical exchange saturation transfer, and quantification of circulating biomarkers^4^.

## Can cell therapy eliminate nociceptive nerve fibers?

An increase in innervation is associated with pain of disc origin^46^. The mechanism of nerve growth and hyperinnervation of pathologically painful disc has not been fully clarified. The molecules that may be involved in this process are some members of the neurotrophin (NTs) family, especially nerve growth factor (NGF), which is known to have neurotrophic and neurotropic properties and regulate the density and distribution of nerve fibers in degenerate disc. NGF and its receptors are expressed in healthy intervertebral discs, but higher levels are observed in painful discs, indicating a correlation between the expression level of NGF and the innervation density of intervertebral discs^46,47^. In addition, anti-NGF therapy has been shown to induce analgesia and show efficacy in patients with chronic low back pain^48^.

A major disadvantage is that no studies to date have been conducted to address the effects of MSCs delivered to the painful disc on ectopic sensory nerve distribution characteristics. Few studies have evaluated the interaction between the delivered cells and the native disc microenvironment, especially with regard to the innervated disc. Strong evidence supports the anti-inflammatory effect of delivery cells in the degenerate discs^8^. However, more and more other applied studies have shown that the regenerative ability of MSCs in neurological diseases, and the NGF and its receptors released by MSCs can enhance nerve survival and neurite outgrowth^49–52^. Therefore, delivery of MSCs to patients with innervated discs may aggravate pain symptoms by supporting the ingrowth of these nociceptive fibers. So far, in the published clinical trials of disc cell therapy, there is always a group of ineffective patients; However, it is unclear why these patients did not show any improvement in pain and disability scores. One possible cause of persistent pain in patients with innervated disc is the survival and migration of neural network mediated by stem cells after delivery^53^.

## Can the delivered exogenous cells survive within degenerate disc?

The nutrient supply of the intervertebral disc cells is mainly through the cartilage endplate. As the intervertebral disc degenerates, endplate calcification may occur and inhibit the diffusion of solutes from the sub-endplate capillary network to the intervertebral disc. In degenerate discs, this nutritional route is hindered, affecting the activity and survival of the implanted cells^9,11^. A large number of animal studies and limited human studies have found that cells remain survive weeks and months after cell transplantation, but few studies have explored longer-term results^54,55^. However, disc repair is a very lengthy process. Type II collagen from healthy mature individuals has a long turnover time, on the order of hundreds of years. This means that the ability of the transplanted cells to produce and secrete the correct matrix components is insufficient for long-term function, as the biomechanically indispensable architectural structures are difficult to form^56^.

Cell therapy that significantly increases the anabolic activity of cells in the degenerate disc and thus increases the nutrient demand will over imbalance the nutritional environment, resulting in cell death or decreased cell activity. Therefore, cell therapy is likely to reduce rather than enhance the activity and viability of delivered cells. Blindly pursuing the intervertebral disc repair strategy to promote cell proliferation and anabolic activity without considering the nutritional environment in the degenerate disc may not be the correct direction of intervertebral disc regeneration. Considering the reduced availability of nutrients during disc degeneration and the importance of adequate nutrition for cell survival, this effect should be considered first when designing disc regeneration strategies^5,11^. Although, more recently, the silicon model study of the in vivo nutrient microenvironment of degenerative intervertebral disc has been found nutrient concentrations may increase by a reduction in diffusional distance due to reduced disc height and vasculature ingrowth^57^. Furthermore, 2-D steady state finite element mathematical modeling research also demonstrated distinct disc morphologies has an important influence on the diffusion gradient of intervertebral disc and found the effect of transplanted cells on nutrition may be limited, with some considerations on dosage^58^. However, the main limitation of these studies is the pure diffusion hypothesis, which still needs to be confirmed by further research. In addition, cell therapy can also produce various angiogenic factors, including vascular endothelial growth factor and fibroblastic growth factor, to promote angiogenesis, which is also essential for the disc repair^8,53^.

## Can cell therapy regenerate degenerate discs?

Mechanical loading of the disc initiates cell-mediated remodeling events that lead to disc degeneration^59,60^. A large number of animal models use altered biomechanics to induce disc degeneration^61^. These models show that although the disc is intact, over time, the altered biomechanical loading can lead to catabolic cell responses and remodeling of the disc matrix. In vitro studies showed that moderate cyclic loading had anabolic effect on disc cells, while static overloading showed catabolic effect^62^. High tension strain applied to human disc cells in vitro has been shown to drive cytokines and inflammatory responses related to intervertebral disc degeneration^63^. Therefore, the relationship between mechanical loading and cell function is considered to be a key component of intervertebral disc function and dysfunction.

The interaction of cells, ECM and biomechanical stress contributes to the homeostasis of the intervertebral disc. If the disc cells do not receive the appropriate mechanical signals, they will stop production and even begin to degrade proteoglycans. The reduction of proteoglycans will cause the pressure in the intervertebral disc to drop, which will change the biomechanical pressure of the cells. Therefore, the positive feedback loop of intervertebral disc degeneration can be deduced, which includes the degenerative cycle of cells, ECM and biomechanics^64^.

Intervertebral disc degeneration is always accompanied by a decrease in pressure within the disc and abnormal load distribution. Under these mechanical conditions, cell therapy to regenerate degenerate discs is almost impossible. From a biomechanical point of view, disc regeneration may only occur under conditions of restoration of pressure and load distribution within the disc. Dynamic stabilization systems now offer the potential for mechanical approaches to disc regeneration. Dynamic stabilization systems using pedicle screws or interspinous devices have shown restabilization of spinal segments and reduction of intradiscal pressure^61^. Numerous clinical studies have shown that degenerative discs receiving dynamic stabilization systems lead to disc regeneration^65^. Combining disc cell-based therapy with a dynamic stabilization system may be the future development direction for the treatment of intervertebral disc degeneration.

## Conclusions

Although cell-based therapy for disc degeneration has made considerable progress, there are still quite a few hurdles to overcome. In this comment, we present major concerns and possible solutions in cell therapy for disc degeneration (Table 2). Further research is needed to develop new tools for diagnosing painful discs that require cell therapy, to develop alternative pathways to eliminate nociceptors growing in painful discs, to further understand the fate and action mechanism of transplanted cells, and to restore the mechanical environment of degenerate discs, so that cell therapy finally moves from the laboratory to the clinic.

**Table 2:** Main limitations and potential solutions of cell therapy for intervertebral disc degeneration.

	Main limitations	Potential solutions
1	Patient selection	Advanced MRI techniques and quantification of circulating biomarkers.
2	Supporting growth of nociceptive nerve fibers in painful discs	Additional therapeutic routes such as intradiscal injection of anti-NGF antibody may be required.
3	Difficult to maintain long-term viability and activity	The development of new technologies, ranging from sophisticated differentiation methods to genome editing, can greatly improve viability and activity of transplanted cells.
4	Inability to restore mechanical loading of intervertebral disc	Cell transplantation combined with dynamic stabilization system.

### Reporting summary

Further information on research design is available in the Nature Research Reporting Summary linked to this article.

## Supplementary information

REPORTING SUMMARY

## Data Availability

Data sharing not applicable to this article as no datasets were generated or analysed during the current study.
